# All’s Bad That Ends Bad: There Is a Peak-End Memory Bias in Anxiety

**DOI:** 10.3389/fpsyg.2019.01272

**Published:** 2019-06-12

**Authors:** Ulrich W. D. Müller, Cilia L. M. Witteman, Jan Spijker, Georg W. Alpers

**Affiliations:** ^1^ Department of Psychology, School of Social Sciences, University of Mannheim, Mannheim, Germany; ^2^ Behavioural Science Institute, Radboud University, Nijmegen, Netherlands

**Keywords:** anxiety, memory bias, recall bias, peak-end bias, exposure

## Abstract

The peak-end memory bias has been well documented for the retrospective evaluation of pain. It describes that the retrospective evaluation of pain is largely based on the discomfort experienced at the most intense point (peak) and at the end of the episode. This is notable because it means that longer episodes with a better ending can be remembered as less aversive than shorter ones; this is even if the former had the same peak in painfulness and an overall longer duration of pain. Until now, this bias has not been studied in the domain of anxiety despite the high relevance of variable levels of anxiety in the treatment of anxiety disorders. Therefore, we set out to replicate the original studies but with an induction of variable levels of anxiety. Of 64 women, half watched a clip from a horror movie which ended at the most frightening moment. The other half watched an extended version of this clip with a moderately frightening ending. Afterward, all participants were asked to rate the global anxiety which was elicited by the video. When the film ended at the most frightening moment, participants retrospectively reported more anxiety than participants who watched the extended version. This is the first study to document that the peak-end bias can be found in the domain of anxiety. These findings require replication and extension to a treatment context to evaluate its implications for exposure therapy.

## Introduction

Several cognitive heuristics and biases have been identified in the past decades. Among the most prominent ones are the representativeness and the availability heuristic (Tversky and Kahneman, 1974), and the attention bias (Phillips et al., 2014). With respect to biases in clinical populations, it is evident that efforts have been made to modify them with training programs (Beard, 2011). Cognitive bias modification (CBM) aims to lessen cognitive biases to treat individuals with alcohol addictions (Eberl et al., 2013), anxiety disorders and depression (Hallion and Ruscio, 2011). There are only a few exceptions in which clinical practice has aimed at capitalizing on cognitive biases. A notable exception is the peak-end bias.

The peak-end bias describes that retrospective evaluations are often dominated by the average discomfort of the worst and the final moments; these are labeled the peak and the end respectively (Kahneman et al., 1993). The peak-end bias has also been referred to as the peak-end rule (e.g., Kemp et al., 2008) or the peak-end effect (e.g., Rode et al., 2007). The phenomenon is closely related to duration neglect (Kahneman et al., 1993). Duration neglect implies that, in general, the duration of an aversive experience only plays a small role in the retrospective evaluation of that experience. Interestingly, the peak-end bias can be utilized as a means to reduce the extent of pain that is remembered. It is shown that this bias can be used in medicine to manipulate the subjective level of pain that is reported by patients, retrospectively after surgery (Redelmeier and Kahneman, 1996; Redelmeier et al., 2003). This is done by prolonging the procedure with a period of less intense pain, instead of stopping when the procedure would normally be over. This is sometimes also referred to as “adding a better ending” (Kahneman et al., 1993). Remarkably, the longer experience is perceived as less painful even though it includes more pain in total, but ends with a period of less intense pain.

Kahneman (2011) gives a possible explanation for the peak-end bias from an evolutionary perspective, regarding which moments are selected for retrospective judgments. He makes a distinction between two selves of humans: “the experiencing self” that does the living and the “remembering self” who keeps score and makes choices. So when he considers these two selves in the context of his research on the peak-end bias and pain, Kahneman states that “the experiencing self” goes through more pain in total while the “remembering self” judges the longer experience retrospectively as less painful. The evaluations made by the “remembering self,” are dominated by the most extreme moment of an experience and hence our choices are influenced by this. He argues that this could have the function to avoid moments that could potentially elicit post-traumatic stress. In line with this argument, Fredrickson (2000) suggested that moments of peak affect receive more weight in the global evaluation because they convey the personal capacity necessary for coping with the experience again. She extended the argument to the end moment, because the end moment determines the boundaries of the experience and thereby defines the peak (Fredrickson, 2000).

Research has shown that the peak-end bias not only influences temporary pain, but also the perception of chronic pain (Stone et al., 2000). Other research has indicated that there is also a peak-end bias for experiences that differ from pain. This includes the perceived unpleasantness of aversive sounds (Schreiber and Kahneman, 2000), aversive video clips (Fredrickson and Kahneman, 1993), impairments of picture quality while watching videos (Hands and Avons, 2001), the somatic symptom breathlessness (Bogaerts et al., 2012; Walentynowicz et al., 2015), and mental effort (Finn, 2010; Finn and Miele, 2016). This implies that the peak-end bias is not limited to experiences of pain.

Another line of research demonstrated that the peak-end bias also has an influence on the subjective perception of pleasure and enjoyment; food is remembered as more pleasant if the more favored component is eaten last compared to when it is eaten first (Robinson et al., 2011). Furthermore, two gifts give more pleasure when the better one is given last compared to when it is given first (Do et al., 2008), and payment sequences (using real payment) with lower end losses are more attractive despite a higher total loss (Langer et al., 2005).

Taken together, the different research-lines support the idea that the peak-end bias is not domain specific, but may be a more general phenomenon. Yet, the domains in which it has been examined are still limited. We set out to examine the peak-end bias in the domain of anxiety due to the clinical significance of implications regarding variable levels of anxiety, and for the potential findings relating to the treatment of anxiety-related disorders. Eliciting anxiety in a systematic manner in the context of exposure is one of the interventions that cognitive behavioral therapy for anxiety disorders relies on most heavily (Craske et al., 2014). Therefore, investigating the peak-end bias in anxiety could be highly informative for the structuring of exposure sessions.

To this end, we induced anxiety in students by exposing them to episodes of a horror movie to examine peak-end effects. We expected that participants in the condition with a less intense level of anxiety at the end would report less anxiety retrospectively than participants in the condition with the peak level of anxiety at the end.

## Materials and Methods

### Participants and Design

In this study, 64 female students of Radboud University participated. They were randomly assigned to one of two conditions (horror movie sequence ending with peak level of anxiety/movie sequence ending with moderate level of anxiety). Only women were invited to participate in this experiment since they are two to three times more likely to be affected by anxiety disorders (Wittchen et al., 2011). Another reason for only inviting woman was that we wanted to keep the sample more homogenous and we did not plan to investigate gender differences in this study. The mean age of the participants was 22.06 years (between the ages of 18 and 37 years). Fifty-four participants were native speakers of Dutch while 10 had a different mother tongue.

### Materials and Apparatus

In the experiment, we used an episode of the horror movie *The Strangers* (Bertino and Davison, 2008). The episode predominantly consists of uncertain threat. It does not contain any aggressive or shocking scenes. The chosen episode was validated beforehand in a pilot study^1^. Based on the plot, the movie episode has a peak (high anxiety-eliciting part), which is followed by a part that only elicits moderate anxiety. The episode was cut after the peak to create two sequences with different endings for the two conditions. The condition ending with the peak was 10 min and 28 s long and the condition ending with the moderate anxiety-eliciting part was 13 min and 28 s long. Furthermore, we used a 15-min clip from the nature documentary *OCEANS* (Perrin, 2009) and a 2-min clip from the comedy film *Despicable Me* (Meledandri and Coffin, 2010). The presentation of the experiment and registration of responses were controlled in the Inquisit software package (Millisecond Software, Seattle, USA).

State anxiety was measured *via* the short-form of the state scale of the Spielberger State-Trait Anxiety Inventory (STAI) (Marteau and Bekker, 1992). This questionnaire consists of six statements that are answered on a 4-point Likert scale ranging from 0 = “absolutely not” to 4 = “very much.” One example is: “I feel calm.” General interest in horror movies was measured with one question: “How much do you like horror movies in general?” Answers are given on a 5-point Likert scale from 0 = “not at all” to 5 = “very much.” To measure trait anxiety, the trait scale of the STAI (Spielberger et al., 1970) was administered, and a quick Big Five questionnaire was used to measure personality (Vermulst and Gerris, 2005). The quick Big Five questionnaire measures personality based on the five-factor model of personality (Goldberg, 1993). The questionnaire consists of a list of 30 adjectives about general personality traits. The questionnaire asks how much a person thinks that these traits apply to them. Answers are given on a 7-point Likert scale from 0 = “not at all” to 7 “very much.” The five subscales extraversion (*α* = 0.85), agreeableness, (*α* = 0.75), conscientiousness (*α* = 0.87), neuroticism (*α* = 0.83), and openness to experience (*α* = 0.76) are measured with six items each.

### Procedure

The procedure was reviewed and approved by the ethics committee for social sciences (ECSW) at Radboud University in Nijmegen. First, informed written consent was obtained from all participants by the experimenter. After this, the experimenter left the room for the rest of the experiment. The presentation of the experiment and registration of given responses were controlled by the Inquisit software package (Millisecond Software, Seattle, USA). Then, participants were exposed to the horror movie sequences of *The Strangers* (Bertino and Davison, 2008) presented on a computer screen in a dark room. The original sound was played over speakers. In one condition, participants were exposed to the sequence ending with the peak and in the other condition to the extended sequence ending with the moderate frightening ending. Apart from this difference in conditions, the procedure was the same for all participants. Directly after watching the sequences, the participants filled in the state anxiety questionnaire (Marteau and Bekker, 1992) to check for a difference in anxiety levels between the conditions.

Next, they were asked if they were familiar with the movie, about their general interest in horror movies, and their demographics. General interest in horror movies may have had an impact on their reaction to the clip they viewed (Hoffner and Levine, 2005). Afterward, the participants filled in the trait anxiety questionnaire (Spielberger et al., 1970) and the quick Big Five (Vermulst and Gerris, 2005), which were included to check for group differences.

Subsequently, the episode of the nature documentary *OCEANS* (Perrin, 2009) was shown to the participants as a filler task to eliminate possible priming effects; this episode was not expected to elicit significant emotions. Priming could otherwise be elicited because of the different endings of the movie sequences. More precisely, participants in the condition with the peak at the end could indicate that they perceived the movie sequence as more frightening solely due to the recency of their experience of the end moments compared to those participants in the condition with the moderate end. This principle is similar to the peak-end bias; however, the fundamental difference is that priming effects are considered to be influential for only a relatively short period of time. The literature states that media priming does not have an effect beyond durations longer than 20 min after the prime (Roskos-Ewoldsen et al., 2002). In contrast, studies on the effects of the peak-end bias on pain have shown that these effects are robust, even up to time periods of 1 year (Redelmeier and Kahneman, 1996). By presenting the documentary, we made sure that there were at least 20 min (trait anxiety questionnaire, quick Big Five, and documentary) between the manipulation and the retrospective assessment of how frightening the participants perceived the movie sequences.

After the filler task, the participants were asked how frightening they thought the movie sequence was, on a 100-point scale ranging from 0 = “absolutely not” to 100 = “very much.” Using a one-item measure for the global retrospective evaluation is common practice in research on the peak-end effect (e.g., Schreiber and Kahneman, 2000; Rode et al., 2007; Kemp et al., 2008; Robinson et al., 2011). Finally, the episode of the comedy film *Despicable Me* (Meledandri and Coffin, 2010) was presented to the participants to counter the induced anxiety and ensure the participants left the experiment in a better mood.

### Data Preparation

Eight participants were familiar with the movie *The Strangers* (Bertino and Davison, 2008), but did not score significantly different from the other participants, neither on the state anxiety questionnaire nor on how frightening they thought the sequences were (analyses are not reported here). Therefore, we included them in the analyses to sustain more statistical power to detect effects.

### Data Analysis^2^

To check whether the sequences evoked different levels of anxiety at the end in the two conditions, an ANOVA was conducted. We used the mean scores per condition (peak ending/moderate ending) on the state anxiety questionnaire (Marteau and Bekker, 1992) administered immediately after the sequences.

To test whether participants in the condition with a less intense level of anxiety at the end would report less anxiety retrospectively than participants in the condition with a more intense level of anxiety at the end, a second ANOVA was conducted. We used the mean scores per condition (peak ending/moderate ending) from the question concerning how frightening the movie sequence was, 20 min after they had seen it.

In addition, to exclude the possibility of significant group differences between the conditions, a MANOVA was conducted, with the mean scores per condition (peak ending/moderate ending) on the trait anxiety questionnaire (Spielberger et al., 1970), the question about enjoyment of horror movies in general and on the dimensions of the personality questionnaire (Vermulst and Gerris, 2005).

To control for individual differences regarding the trait anxiety of the participants, we ran an ANCOVA with the mean score per condition (peak ending/moderate ending) on the state anxiety questionnaire and as a covariate, the scores on the trait anxiety questionnaire.

Furthermore, we ran an ANCOVA with the mean scores per condition on the question how frightening the movie sequence was retrospectively and as a covariate, the scores on the trait anxiety questionnaire.

To explore the influence of individual differences in the preference of horror movies, we ran an ANCOVA with the mean scores per condition (peak ending/moderate ending) on the state anxiety questionnaire and as a covariate, the scores on the question regarding how much participants liked horror movies in general.

Moreover, we controlled for the effect of how much participants liked horror movies in general on the effect between conditions of how frightening the participants thought the horror movie sequence had been 20 min after they saw it. Therefore, we ran an ANCOVA with the mean scores per condition (peak ending/moderate ending) on the retrospective recall of the levels of anxiety evoked by the horror movie sequences and as covariate the scores on the question regarding how much participants liked horror movies in general.

## Results

### Manipulation Check

**Figure 1:**
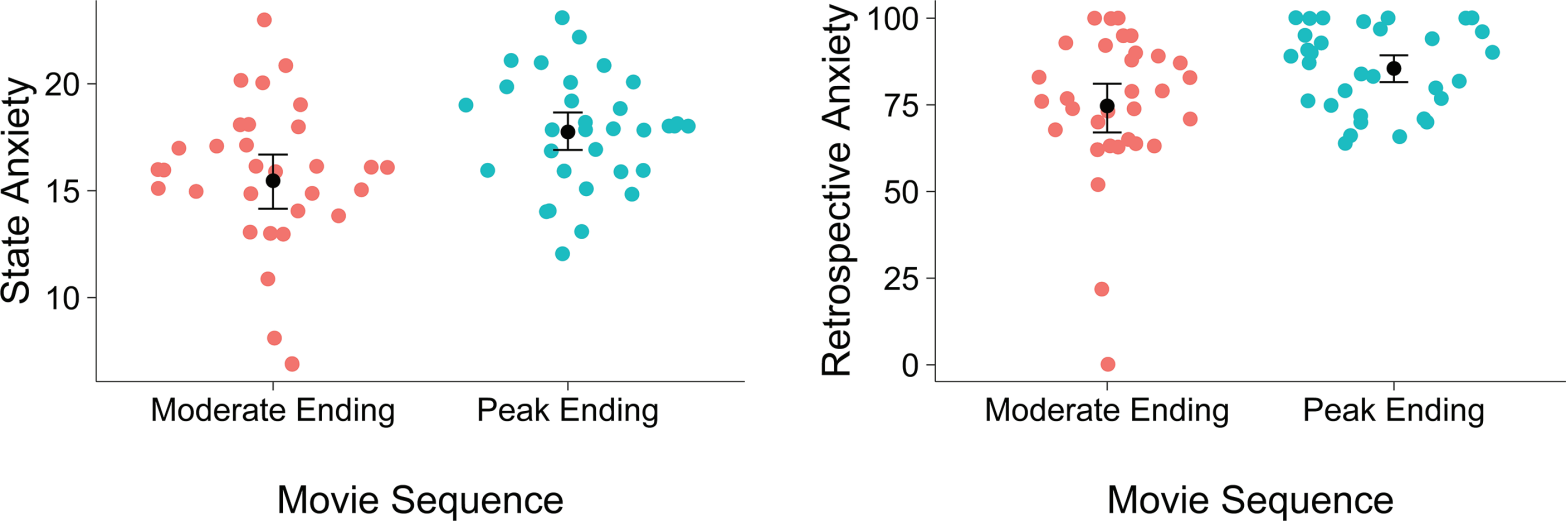
**Left panel**: Sinaplot (Sidiropoulos et al., 2017) showing the original data (see text footnote 2), means and confidence intervals of the reported state anxiety directly after the horror movie sequences separately for the condition with the moderate end and the condition ending with the peak. **Right panel**: Sinaplot showing the original data (see text footnote 2), means and confidence intervals of the reported retrospective anxiety 20 min after the presentation of the horror movie sequences separately for the condition with the moderate end and the condition ending with the peak.

As intended, the participants who had been exposed to the sequence ending with the peak were more anxious directly after watching the sequence (*M* = 17.7, *SD* = 2.60) than the participants who were exposed to the sequence with the moderate ending (*M* = 15.5, *SD* = 3.64); *F*(1,62) = 8.30, *p =* 0.005; *η*^2^ = 0.118. This effect was in the medium range. See also Figure 1.

### Control for Group Differences

We did not find any significant differences between the conditions (peak ending/moderate ending) on the trait anxiety questionnaire, on any of the five dimensions of the personality questionnaire and on enjoyment of horror movies in general; *F*(7, 56) = 1.67, *p* = 0.135. See also Table 1.

**Table 1:** Means, standard deviations and MANOVA comparisons for the questionnaires scores of the two conditions (peak anxiety at the end of the sequence *n* = 32, moderate anxiety at the end of the sequence *n* = 32).

Questionnaire	Peak ending	Moderate ending	MANOVA		
	*M* (SD)	*M* (SD)	*F*	df	*p*
STAI-T	40.41 (9.88)	35.78 (10.12)	3.42	62	0.069
N	23.75 (6.17)	24.03 (6.75)	0.03	62	0.862
E	24.47 (5.95)	25.06 (6.32)	0.15	62	0.700
O	28.53 (6.02)	29.50 (5.54)	0.45	62	0.506
C	29.28 (6.65)	26.50 (6.70)	2.78	62	0.101
A	34.59 (3.28)	24.37 (3.19)	0.07	62	0.788
Enjoyment	1.56 (0.91)	1.88 (1.10)	1.53	62	0.221

*STAI-T, State-Trait Anxiety Inventory-Trait form (Spielberger et al., 1970); N, Neuroticism; E, Extraversion; O, Openness to experience; C, Conscientiousness; A, agreeableness (quick Big Five questionnaire, Vermulst and Gerris, 2005); Enjoyment: Question about enjoyment of horror movies in general*.

### Peak-End Bias

After watching the filler, the retrospective recall of the levels of anxiety evoked by the horror movie sequences differed between the conditions. In line with our hypothesis, participants who had been exposed to the sequence ending with the peak retrospectively reported that they thought the sequence was more frightening (*M* = 85.5, *SD* = 11.98) than the participants who had been exposed to the sequence with the moderate ending (*M* = 74.69, *SD* = 21.30); *F*(1, 62) = 6.26, *p* = 0.015; *η*^2^ *=* 0.092. This effect was in the medium range. See also Figure 1.

### Control for Individual Differences

Trait anxiety was not a significant covariate in how much anxiety the participants reported directly after watching the horror movie sequence in the two conditions; *F*(1, 61) = 2.70, *p* = 0.105; trait anxiety did not influence the effect of the different movie sequences on state anxiety at the end of the sequences; *F*(1, 61) = 6.09, *p* = 0.016; *η*^2^ = 0.091. Furthermore, trait anxiety was not a significant covariate for the retrospective recall of the level of anxiety elicited by the horror movie sequences; *F*(1, 61) = 1.03, *p* = 0.314; including trait anxiety as covariate did not alter the found peak-end effects; *F*(1, 61) = 4.85, *p* = 0.031; *η*^2^ = 0.074.

Enjoyment of horror movies in general was negatively related to how anxious the participants were directly after watching the horror movie sequence; *F*(1, 61) = 10.68, *p* = 0.002; *η*^2^ = 0.149; this effect was strong. Nonetheless, controlling for the enjoyment of horror movies in general did not significantly impact the effect of the different movie sequences on the state anxiety at the end of the sequences; *F*(1, 61) = 6.52, *p* = 0.013; *η*^2^ = 0.097. Enjoyment of horror movies in general was negatively related to the retrospective recall of the level of anxiety elicited by the horror movie sequences; *F*(1, 61) = 15.39, *p* < 0.001; *η*^2^ = 0.201; this effect was strong. However, controlling for the enjoyment of horror movies in general did not significantly affect the effect of the different movie sequences on the retrospective recall of the level of anxiety; *F*(1, 61) = 4.56, *p* = 0.037; *η*^2^ = 0.070.

## Discussion

This is the first study to examine the peak-end bias in the domain of anxiety. It clearly demonstrates that in retrospect, a frightening experience is rated as less frightening if it does not end with the most poignant moment. The experience is rated less frightening although it has the same peak and an additional anxiety-eliciting component, thereby including more anxiety in total. This is paradoxical because a more objective evaluation would result in the opposite judgment.

In the experiment, we found peak-end effects in students watching the movie sequences. Earlier research has shown that the peak-end bias applies to a range of retrospective judgments about acute pain (Kahneman et al., 1993), chronic pain (Stone et al., 2000), aversive picture quality (Hands and Avons, 2001), aversive sounds (Schreiber and Kahneman, 2000), and pleasure and enjoyment (Langer et al., 2005; Do et al., 2008; Robinson et al., 2011). The hypothesis, based on the earlier research, was that the peak-end bias is a universal phenomenon of retrospective evaluations and therefore also applies to anxiety. More specifically, we expected that participants in the condition with a less intense level of anxiety at the end would report less anxiety retrospectively than those participants in the condition with the peak level of anxiety at the end. This hypothesis was confirmed.

The state anxiety questionnaire (Marteau and Bekker, 1992) confirmed the different intensities of anxiety between the conditions directly after the exposure to the sequences. It was also ascertained through multiple filler tasks that at least 20 min passed between the manipulation and the assessment of the retrospective anxiety. This was to ensure that the results could not be compromised by priming effects (e.g., Roskos-Ewoldsen et al., 2002). Thereby, the peak-end effect can clearly be attributed to the difference in the conditions.

Our research confirms that the peak-end bias can also be observed in the domain of anxiety; this supports the notion that the peak-end effect is not domain specific but a general phenomenon of human retrospective evaluations.

Considering individual differences, we found that trait anxiety of the participants did not affect the found peak-end effects. Regarding the anxiety induction by means of horror movie sequences, we found that participants that enjoy horror movies in general were less anxious at the end of the horror movie sequences. In line with this finding, peak-end effects were stronger in participants that do not enjoy horror movies in general. This is an artifact of the used anxiety induction. For persons that enjoy horror movies, anxiety in the context of horror movies has not a clear positive or negative valence. They might enjoy being anxious in this context.

However, this study has certain limitations. Some of them originate from our decision to follow the original peak-end paradigm as closely as possible which we adopted to the domain of anxiety for the first time. First, there is only a single item for the global retrospective evaluation. This might limit psychometric quality, but such a measure was successful in detecting the effects of the manipulation in previous research on the peak-end bias (e.g., Schreiber and Kahneman, 2000; Rode et al., 2007; Kemp et al., 2008; Robinson et al., 2011) and it was sensitive in the present study. Second, the movie sequences differed in length; the sequence with the less frightening ending was somewhat longer. However, this again is common in research on the peak-end bias (e.g., Redelmeier and Kahneman, 1996; Redelmeier et al., 2003). Importantly, other research shows that the peak-end effect also holds for experiences of equal duration (e.g., Stone et al., 2000; Hands and Avons, 2001). In one aspect, we deviated from the original paradigm. We did not continuously collect ratings of anxiety during the presentation of the sequences. We did not do so because this could distract from the movie sequence and distraction could impede the induction of anxiety (Foa and Kozak, 1986). Nonetheless, we validated the sequence beforehand in a pilot study^1^ with continuous heart rate measurements in a separate sample.

More specifically, a horror movie might not be the optimal way to induce anxiety in the context of the peak-end bias for two reasons. First, horror movies might have a different valence for different participants; some may enjoy the thrill. However, the presentation of such movies is well established in the literature and it is one of the most common and effective methods of emotion elicitation; it is generally thought to be valid to induce anxiety (Schaefer et al., 2010). Also, our results demonstrate that controlling for general enjoyment of horror movies does not significantly impact the peak-end bias we found. Second, in contrast to the manipulations used in the studies on pain, the movie clips contain a plot. The plot consists of a specific threat that varied in intensity over time but was continuously present and never resolved. It is difficult to rule out that the additional information provided in the longer sequence resulted in reappraisal of the content of the shorter one. In any case, such a perspective does not argue against the existence of the peak-end bias in anxiety. Instead, it might reflect on its underlying mechanism.

Furthermore, as in all research with university students, the sample might not be representative. On the one hand, we tested only women in this experiment since they are two to three times more likely to be affected by anxiety disorders (Wittchen et al., 2011) and to keep the sample more homogenous. On the other hand, for ethical reasons, participants were informed that highly emotional material would be shown to them during the experiment, which might have led to self-selection bias in participation. These participants may be more emotionally stable than the general population (Almeida et al., 2008). However, we did not find any indication for a floor effect in the trait anxiety measure. On the other hand, the peak-end bias we found may be a conservative account; it may be considerably stronger in patients with anxiety-related psychopathology.

To address the limitations of the study, we suggest that future studies use a manipulation to elicit fear which does not have ambiguous valence and does not have a plot. Anxiety could be elicited in a (clinical) sample that is afraid of certain stimuli by exposing participants to their fear, for example fear of heights (e.g., Davis et al., 2011) or fear of spiders (e.g., Gerdes et al., 2009). Furthermore, such a preselection based on specific fears could also eliminate the possibility self-selection bias in participation. In addition, given the differences in prevalence of anxiety disorders between men and woman, future studies should also investigate potential gender differences in the context of the peak-end bias in anxiety.

It is an open question whether a less aversive retrospective memory of anxiety is predictive of future behavior as it was demonstrated in the domain of pain (Kahneman et al., 1993). This would be especially interesting for clinical practice in the context of avoidance behavior and dropout rates which are common in anxiety disorders. Future research should investigate the peak-end bias in a clinical population. One of the most effective treatments for anxiety-related disorders is exposure therapy (Powers et al., 2010; Ougrin, 2011), in which patients are exposed to the objects of their pathological fears. Studying the peak-end memory bias in this context could be highly informative for clinicians and for the design of exposure therapy. Anxiety typically peaks in the beginning of exposure and wanes within and across sessions (Alpers et al., 2005; Alpers and Sell, 2008). The exposure itself is often thought to require a noticeable reduction of anxiety to be effective (Foa and Kozak, 1986; Alpers, 2010; Gloster et al., 2011); however, other researchers have stated that this is not necessary (Craske et al., 2014). In any case, the aversive memory of anxiety experienced during exposure may be less aversive if exposure lasted until anxiety waned.

Given the required replication of our results in a clinical sample, we recommend to devote special attention to the structuring of exposure therapy in order to facilitate a noticeable anxiety reduction toward the end (compared to the peak anxiety) within one exposure session. Even if the literature is not entirely conclusive on whether such fear reduction is necessary for the effectiveness of exposure, it may nonetheless render the patient’s memory of the experience as less aversive. Another desirable effect may be that fewer patients may drop out because a less aversive memory could reduce avoidance behavior of similar experiences. Better understanding of this bias may help to reduce dropout rates which are a common problem in exposure-based treatments (e.g., Belleau et al., 2017).

## Ethics Statement

The procedure was reviewed and approved by the ethics committee for social sciences (ECSW) at Radboud University in Nijmegen.

## Author Contributions

UM conceived and designed the study, conducted the data acquisition, analysis, and interpretation of the results, and also drafted the work. CW and JS supervised data collection. GA, CW, and JS contributed to the interpretation of the results and writing of the paper.

### Conflict of Interest Statement

The authors declare that the research was conducted in the absence of any commercial or financial relationships that could be construed as a potential conflict of interest.

## Footnotes

^1^An independent sample of 41 female students participated in the pilot study. Statistical analysis of the heart rate confirmed that the heart rate increased during exposure to the complete movie sequence compared to a baseline measurement. We found that the sequence continuously elicited a significantly higher heart rate compared to the baseline measurement. We did not collect self-reported anxiety ratings or a state anxiety questionnaire in the pilot study.

^2^Some of the dependent variables are not normally distributed and have outliers; therefore, the analyses were also conducted without outliers and on transformed data to account for violations of assumptions. Since this did not substantially change any of the results, we report the analyses and show graphs of the original data.

## References

[ref1] Almeida L. Kashdan T. B. Nunes T. Coelho R. Albino-Teixeira A. Soares-da-Silva P. (2008). Who volunteers for phase I clinical trials? Influences of anxiety, social anxiety and depressive symptoms on self-selection and the reporting of adverse events. Eur. J. Clin. Pharmacol. 64, 575–582. 10.1007/s00228-008-0468-8, PMID: 18320183

[ref2] Alpers G. W. (2010). “Avoiding treatment failures in specific phobias” in Avoiding treatment failures in the anxiety disorders (series in anxiety and related disorders). eds. Otto M. W. Hofmann S. G. (New York: Springer).

[ref3] Alpers G. W. Sell R. (2008). And yet they correlate: psychophysiological activation predicts self-report outcomes of exposure therapy in claustrophobia. J. Anxiety Disord. 22, 1101–1109. 10.1016/j.janxdis.2007.11.009, PMID: 18164177

[ref4] Alpers G. W. Wilhelm F. H. Roth W. T. (2005). Psychophysiological assessment during exposure in driving phobic patients. J. Abnorm. Psychol. 114, 126–139. 10.1037/0021-843X.114.1.126, PMID: 15709819

[ref5] Beard C. (2011). Cognitive bias modification for anxiety: current evidence and future directions. Expert. Rev. Neurother. 11, 299–311. 10.1586/ern.10.194 21306216 PMC3092585

[ref6] Belleau E. L. Chin E. G. Wanklyn S. G. Zambrano-Vazquez L. Schumacher J. A. Coffey S. F. (2017). Pre-treatment predictors of dropout from prolonged exposure therapy in patients with chronic posttraumatic stress disorder and comorbid substance use disorders. Behav. Res. Ther. 91, 43–50. 10.1016/j.brat.2017.01.011, PMID: 28147254 PMC5328858

[ref7] Bertino B. Davison D (2008). The Strangers [Motion Picture]. (United States: Rogue).

[ref8] Bogaerts K. Wan L. Diest I. V. Stans L. Decramer M. Van den Bergh O. (2012). Peak-end memory bias in laboratory-induced dyspnea: a comparison of patients with medically unexplained symptoms and healthy controls. Psychosom. Med. 74, 974–981. 10.1097/PSY.0b013e318273099c, PMID: 23115343

[ref9] Craske M. G. Treanor M. Conway C. C. Zbozinek T. Vervliet B. (2014). Maximizing exposure therapy: an inhibitory learning approach. Behav. Res. Ther. 58, 10–23. 10.1016/j.brat.2014.04.006, PMID: 24864005 PMC4114726

[ref10] Davis J. R. Horslen B. C. Nishikawa K. Fukushima K. Chua R. Inglis J. T.. (2011). Human proprioceptive adaptations during states of height-induced fear and anxiety. J. Neurophysiol. 106, 3082–3090. 10.1152/jn.01030.2010, PMID: 21918000

[ref11] Do A. M. Rupert A. V. Wolford G. (2008). Evaluations of pleasurable experiences: the peak-end rule. Psychon. Bull. Rev. 15, 96–98. 10.3758/PBR.15.1.96, PMID: 18605486

[ref12] Eberl C. Wiers R. W. Pawelczack S. Rinck M. Becker E. S. Lindenmeyer J. (2013). Approach bias modification in alcohol dependence: do clinical effects replicate and for whom does it work best? Dev. Cogn. Neurosci. 4, 38–51. 10.1016/j.dcn.2012.11.002, PMID: 23218805 PMC6987692

[ref13] Finn B. (2010). Ending on a high note: adding a better end to effortful study. J. Exp. Psychol. Learn. Mem. Cogn. 36, 1548–1553. 10.1037/a0020605, PMID: 20854005 PMC2970645

[ref14] Finn B. Miele D. B. (2016). Hitting a high note on math tests: remembered success influence test preferences. J. Exp. Psychol. Learn. Mem. Cogn. 42, 17–38. 10.1037/xlm0000150, PMID: 26213835

[ref15] Foa E. B. Kozak M. J. (1986). Emotional processing of fear: exposure to corrective information. Psychol. Bull. 99, 20–35. 10.1037/0033-2909.99.1.20, PMID: 2871574

[ref16] Fredrickson B. L. (2000). Extracting meaning from past affective experiences: the importance of peaks, ends, and specific emotions. Cognit. Emot. 14, 577–606. 10.1080/026999300402808

[ref17] Fredrickson B. L. Kahneman D. (1993). Duration neglect in retrospective evaluations of affective episodes. J. Pers. Soc. Psychol. 65, 45–55. 10.1037/0022-3514.65.1.45, PMID: 8355141

[ref18] Gerdes A. B. M. Uhl G. Alpers G. W. (2009). Spiders are special: fear and disgust evoked by pictures of arthropods. Evol. Hum. Behav. 30, 66–73. 10.1016/j.evolhumbehav.2008.08.005

[ref19] Gloster A. T. Wittchen H.-U. Einsle F. Lang T. Helbig-Lang S. Fydrich T.. (2011). Psychological treatment for panic disorder with agoraphobia: a randomized controlled trial to examine the role of therapist-guided exposure in situ in CBT. J. Consult. Clin. Psychol. 79, 406–420. 10.1037/a0023584, PMID: 21534651

[ref20] Goldberg L. R. (1993). The structure of phenotypic personality traits. Am. Psychol. 48, 26–34. 10.1037/0003-066X.48.1.26, PMID: 8427480

[ref21] Hallion L. S. Ruscio A. M. (2011). A meta-analysis of the effect of cognitive bias modification on anxiety and depression. Psychol. Bull. 137, 940–958. 10.1037/a0024355, PMID: 21728399

[ref22] Hands D. S. Avons S. E. (2001). Recency and duration neglect in subjective assessment of television picture quality. Appl. Cogn. Psychol. 15, 639–657. 10.1002/acp.731

[ref23] Hoffner C. A. Levine K. J. (2005). Enjoyment of mediated fright and violence: a meta-analysis. Media Psychol. 7, 207–237. 10.1207/S1532785XMEP0702_5

[ref24] Kahneman D. (2011). Thinking, fast and slow. (New York: Farrar, Strauss, Giroux).

[ref25] Kahneman D. Fredrickson B. L. Schreiber C. A. Redelmeier D. A. (1993). When more pain is preferred to less: adding a better end. Psychol. Sci. 4, 401–405. 10.1111/j.1467-9280.1993.tb00589.x

[ref26] Kemp S. Burt C. D. B. Furneaux L. (2008). A test of the peak-end rule with extended autobiographical events. Mem. Cogn. 36, 132–138. 10.3758/MC.36.1.132, PMID: 18323069

[ref27] Langer T. Sarin R. Weber M. (2005). The retrospective evaluation of payment sequences: duration neglect and peak-and-end effects. J. Econ. Behav. Organ. 58, 157–175. 10.1016/j.jebo.2004.01.001

[ref28] Marteau T. M. Bekker H. (1992). The development of a six-item short-form of the state scale of the Spielberger state-trait anxiety inventory (STAI). Br. J. Clin. Psychol. 31, 301–306. 10.1111/j.2044-8260.1992.tb00997.x, PMID: 1393159

[ref29] Meledandri C. Coffin P. (2010). Despicable me [Motion Picture]. (United States: Universal Pictures).

[ref30] Ougrin D. (2011). Efficacy of exposure versus cognitive therapy in anxiety disorders: systematic review and meta-analysis. BMC Psychiatry 11:200. 10.1186/1471-244X-11-200 22185596 PMC3347982

[ref31] Perrin J. (2009). OCEANS [Motion Picture]. (France: Pathé).

[ref32] Phillips K. Wright B. J. Kent S. (2014). Irritable bowel syndrome and symptom severity: evidence of negative attention bias, diminished vigour, and autonomic dysregulation. J. Psychosom. Res. 77, 13–19. 10.1016/j.jpsychores.2014.04.009, PMID: 24913336

[ref33] Powers M. B. Halpern J. M. Ferenschak M. P. Gillihan S. J. Foa E. B. (2010). A meta-analytic review of prolonged exposure for posttraumatic stress disorder. Clin. Psychol. Rev. 30, 635–641. 10.1016/j.cpr.2010.04.007, PMID: 20546985

[ref34] Redelmeier D. A. Kahneman D. (1996). Patients memories of painfull medical treatments: real time and retrospective evaluations of two minimally invasive procedures. Pain 66, 3–8. 10.1016/0304-3959(96)02994-6, PMID: 8857625

[ref35] Redelmeier D. A. Katz J. Kahneman D. (2003). Memories of colonoscopy: a randomized trial. Pain 104, 187–194. 10.1016/S0304-3959(03)00003-4, PMID: 12855328

[ref36] Robinson E. Blissett J. Higgs S. (2011). Peak and end effects on remembered enjoyment of eating in low and high restrained eaters. Appetite 57, 207–212. 10.1016/j.appet.2011.04.022, PMID: 21570432

[ref37] Rode E. Rozin P. Durlach P. (2007). Experienced and remembered pleasure for meals: duration neglect but minimal peak, end (recency) or primacy effects. Appetite 49, 18–29. 10.1016/j.appet.2006.09.006, PMID: 17459522

[ref38] Roskos-Ewoldsen D. R. Roskos-Ewoldsen B. Dillman Carpentier F. R. (2002). “Media priming: a synthesis” in Media effects: Advances in theory and research. eds. Bryant J. Zillmann D. (Mahwah, NJ: Lawrence Erlbaum Associates), 97–120.

[ref39] Schaefer A. Nils F. Sanchez X. Philippot P. (2010). Assessing the effectiveness of a large database of emotion-eliciting films: a new tool for emotion researchers. Cognit. Emot. 24, 1153–1172. 10.1080/02699930903274322

[ref40] Schreiber C. A. Kahneman D. (2000). Determinants of the remembered utility of aversive sounds. J. Exp. Psychol. Gen. 129, 27–42. 10.1037/0096-3445.129.1.27, PMID: 10756485

[ref41] Sidiropoulos N. Sohi S. H. Pedersen T. L. Porse B. T. Winther O. Rapin N. (2017). SinaPlot: an enhanced chart for simple and truthful representation of single observations over multiple classes. J. Comput. Graph. Stat. 27, 673–676. 10.1080/10618600.2017.1366914

[ref42] Spielberger C. D. Gorsuch R. L. Lushene P. R. Jacobs A. G. (1970). State-trait anxiety inventory (test manual). (Palo Alto, CA: Consulting Psychologists Press).

[ref43] Stone A. A. Broderick J. E. Kaell A. T. DelesPaul P. A. E. G. Porter L. E. (2000). Does the peak-end phenomenon observed in laboratory pain studies apply to real-world pain in rheumatoid arthritics? J. Pain 1, 212–217. 10.1054/jpai.2000.7568, PMID: 14622620

[ref44] Tversky A. Kahneman D. (1974). Judgement under uncertainty: heuristics and biases. Science 185, 1124–1131. 10.1126/science.185.4157.1124, PMID: 17835457

[ref45] Vermulst A. A. Gerris J. R. M. (2005). QBF: Quick big five Persoonlijkheidstest Handleiding [Quick big five personality test manual]. (Leeuwarden, the Netherlands: LDC Publications).

[ref46] Walentynowicz M. Bogaerts K. Van Diest I. Raes F. Van den Bergh O. (2015). Was it so bad? The role of retrospective memory in symptom reporting. Health Psychol. 34, 1166–1174. 10.1037/hea0000222 26010720

[ref47] Wittchen H. U. Jacobi F. Rhem J. Gustavsson A. Svensson M. Jönsson B. (2011). The size and burden of mental disorders and other disorders of the brain in europe 2010. Eur. Neuropsychopharmacol. 21, 655–679. 10.1016/j.euroneuro.2011.07.018 21896369

